# Association of Hemoglobin Glycation Index With All-Cause Mortality, Cardiac Mortality, and Cardiovascular Mortality in the General Population: A Retrospective Cohort Study of NHANES Data

**DOI:** 10.31083/RCM36792

**Published:** 2025-07-28

**Authors:** Qing Mao, Jingjing Wang, Shuang Zuo, Liyou Xu, Liu Ji, Haishan Li

**Affiliations:** ^1^Department of Emergency, The Second People’s Hospital of Hefei, Hefei Hospital Affiliated to Anhui Medical University, 230011 Hefei, Anhui, China

**Keywords:** glycated hemoglobin, NHANES, mortality, cardiovascular diseases

## Abstract

**Background:**

The hemoglobin glycation index (HGI) presents a discrepancy between observed and predicted glycosylated hemoglobin (HbA1c) and fasting blood glucose values. Meanwhile, compared to the HbA1c values, the HGI provides a more comprehensive reflection of blood glucose variability across populations. However, no studies have examined the association between the HGI and all-cause, cardiac, and cardiovascular mortalities in the general population. Hence, this study aimed to investigate these relationships using data from the National Health and Nutrition Examination Survey (NHANES) database.

**Methods:**

Participants were stratified into four groups based on the HGI quartiles. Weighted multivariable Cox proportional hazards models were used to assess the associations between HGI and all-cause, cardiovascular, and cardiac mortality. Kaplan–Meier survival analysis based on the HGI quartiles and log-rank tests were employed to compare differences in primary and secondary endpoints. Additionally, restricted cubic spline (RCS) curves were used to explore nonlinear relationships between the HGI and endpoints, identifying inflection points. Subgroup analyses and interaction tests were conducted to assess the robustness of the findings.

**Results:**

In comparing the baseline characteristics of endpoints across all-cause mortality, cardiac mortality, and cardiovascular mortality, significantly higher mortality rates were observed in the high HGI quartile group (Q4) compared to the other three groups (Q1, Q2, and Q3) (*p* < 0.05). Kaplan–Meier curves demonstrated increased mortality risks in the high HGI group across all endpoints (*p* < 0.05). Multivariable Cox proportional hazards models indicated that high HGI levels were associated with all-cause mortality (Q4: hazard ratio (HR) (95% confidence interval (CI)) = 1.232 (1.065, 1.426); *p* = 0.005), cardiac mortality (HR (95% CI) = 1.516 (1.100, 2.088); *p* = 0.011) and cardiovascular mortality (HR (95% CI) = 1.334 (1.013, 1.756); *p* = 0.039). Low HGI was associated only with all-cause mortality (Q1: HR (95% CI) = 1.269 (1.082, 1.488); *p* = 0.003). RCS analysis confirmed a U-shaped relationship between the HGI and all three outcome events. Subgroup analyses and interaction tests supported the robustness of the conclusions.

**Conclusion:**

This study demonstrates a U-shaped association between the HGI and overall mortality, cardiac mortality, and cardiometabolic mortality in the general population. Specifically, the high HGI value represented a risk factor for all-cause, cardiac, and cardiovascular mortality. In contrast, low HGI values were associated only with all-cause mortality in the general population.

## 1. Introduction

Cardiovascular diseases (CVDs), characterized by their high morbidity and mortality rates, pose a significant global health challenge and a substantial burden on healthcare systems worldwide. As populations age, CVDs have emerged as the leading cause of death globally, responsible for nearly one-third of all fatalities, with a marked increase of 12.5% observed over the past decade [1, 2]. CVDs now rank as the foremost contributor to reduced life expectancy among older people [2, 3]. To effectively reduce CVD risk, early identification of individuals at high risk during the progression of cardiovascular disease is crucial.

The hemoglobin glycation index (HGI) is derived from the difference between observed and predicted glycosylated hemoglobin (HbA1c) values. Moreover, the HGI serves as a reliable metric to quantify glucose metabolism and individual variability, having been validated as an effective measure of deviations in HbA1c levels [4]. Although HbA1c remains the gold standard for assessing glycemic control in diabetes, its levels can be influenced by factors such as red blood cell turnover and glucose gradients across the red blood cell membrane, reflecting only 60–80% of the glucose levels found in the body [5]. To address these limitations and provide a more accurate assessment of glycemic variability, Hempe and colleagues [6] introduced the HGI, which has subsequently been demonstrated to improve blood glucose variability capturing in diverse populations [7].

Given that the HGI represents blood glucose variability, prior research has primarily focused on its prognostic implications in diabetic populations. However, studies investigating the correlation between the HGI and overall mortality rates in the general population are scarce [8, 9]. Indeed, recent research indicated a U-shaped relationship between the HGI and all-cause mortality in the general population [10]. However, the association between the HGI and cardiac and cardiovascular mortality in the general population remains unclear.

To our knowledge, no studies have yet investigated the relationship between HGI and cardiometabolic mortality. Therefore, this study aimed to utilize data from the National Health and Nutrition Examination Survey (NHANES) database to conduct a retrospective cohort analysis. Moreover, this study sought to explore the associations and disparities of the HGI with all-cause mortality, cardiac mortality, and cardiovascular mortality in the general population.

## 2. Methods and Materials 

### 2.1 Study Population

The NHANES database is a periodic survey conducted by the National Center for Health Statistics (NCHS) of the United States. The NHANES database systematically examines a random sample of American citizens through comprehensive physical examinations and questionnaires. Indeed, the NHANES collects data on physiological measurements, nutritional status, health surveys, and environmental factors to assess the health and nutritional status of the U.S. population (https://wwwn.cdc.gov/nchs/nhanes/Default.aspx). The NHANES has received approval from the NCHS Research Ethics Review Board to ensure ethical standards in research.

**Fig. 1.**
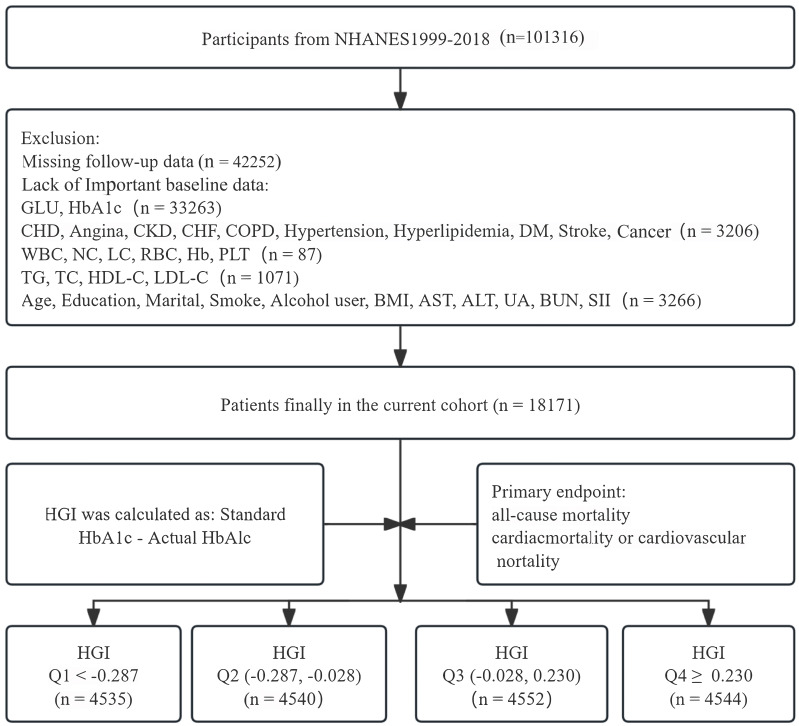
**Flowchart for inclusion of participants**. Abbreviations: NHANES, National Health and Nutrition Examination Survey; GLU, glucose; HbA1c, glycosylated hemoglobin; CHD, coronary heart disease; CKD, chronic kidney disease; CHF, congestive heart failure; DM, diabetes mellitus; WBC, white blood cell count; NC, neutrophil count; LC, lymphocyte count; RBC, red blood cell count; Hb, hemoglobin; PLT, platelet count; TG, triglyceride; TC, total cholesterol; HDL-C, high-density lipoprotein cholesterol; LDL-C, low-density lipoprotein cholesterol; BMI, body mass index; AST, aspartate aminotransferase; ALT, alanine aminotransferase; UA, uric acid; BUN, blood urea nitrogen; SII, systemic immune-inflammation index; HGI, hemoglobin glycation index; COPD, chronic obstructive pulmonary disease; Q1, first quartile; Q2, second quartile; Q3, third quartile; Q4, fourth quartile.

The exclusion criteria included individuals with severe liver or kidney disease, those lacking follow-up data or inadequate follow-ups, individuals missing baseline data such as glycated hemoglobin, fasting glucose, or lipid levels, and those with less than 8 hours of fasting before blood specimen collection. A flowchart outlining the patient selection process is presented in Fig. 1. Following the application of these criteria, our study included 18,171 samples spanning from 1999 to 2018, with participants undergoing up to 10 follow-up assessments over a median follow-up period of 112 months.

### 2.2 Data Collection

Our study incorporated four primary categories of covariates: (1) demographic characteristics, including sex, age, race, smoking status, alcohol consumption, marital status, and education level; (2) general physical measures, such as body mass index (BMI); (3) laboratory parameters encompassing alanine aminotransferase (ALT), aspartate aminotransferase (AST), creatinine (CR), blood urea nitrogen (BUN), triglyceride (TG) level, total cholesterol (TC), high-density lipoprotein cholesterol (HDL-C), low-density lipoprotein cholesterol (LDL-C), uric acid (UA), fasting plasma glucose (FPG), HbA1c, white blood cell count (WBC), neutrophil count (NC), lymphocyte count (LC), platelet count (PLT), and red blood cell count (RBC); (4) medical history including hypertension, coronary artery disease (CAD), angina pectoris, chronic heart failure (CHF), diabetes mellitus (DM), chronic obstructive pulmonary disease (COPD), cancer, chronic kidney disease (CKD), and hyperlipidemia. The HGI was calculated using the formula HGI = observed HbA1c – predicted values (predicted HbA1c values derived from a linear regression equation according to the FPG and observed HbA1c) [11]. All blood specimens were collected following a minimum 8-hour fasting period.

### 2.3 Definition of Exposure Variables and Outcome Events

The primary endpoint of this study was defined as all-cause mortality (cause of death: Alzheimer’s disease (G30), diseases of the heart (I00–I09, I11, I13, I20–I51), chronic lower respiratory diseases (J40–J47), malignant neoplasms (C00–C97), all other causes (residual), cerebrovascular diseases (I60–I69), accidents (unintentional injuries) (V01–X59, Y85–Y86), diabetes mellitus (E10–E14), influenza and pneumonia (J09–J18), nephritis, nephrotic syndrome, and nephrosis (N00–N07, N17–N19, N25–N27)), while the secondary endpoint was defined as cardiac mortality (diseases of the heart (I00–I09, I11, I13, I20–I51)) or cardiovascular mortality (diseases of the heart (I00–I09, I11, I13, I20–I51), cerebrovascular diseases (I60–I69)). Follow-up continued until the time of death.

### 2.4 Statistical Analyses

Statistical analyses were performed using R (version 4.2.3; The R Foundation for Statistical Computing, Vienna, Austria), EmpowerStats (version 4.2; X&Y Solutions, Hangzhou, Zhejiang, China), and GraphPad Prism (version 9.0; GraphPad Software, San Diego, CA, USA). Participants were stratified into four quartiles based on the HGI values: Q1 (HGI <–0.287, n = 4535), Q2 (–0.287 ≤ HGI < –0.028, n = 4540), Q3 (–0.028 ≤ HGI < 0.230, n = 4552), and Q4 (HGI ≥ 0.230, n = 4544). Continuous variables were expressed as medians (interquartile range: P25, P75), and categorical variables were reported as counts (percentages). The normality test was conducted using the Kolmogorov-Smirnov test. Group differences for continuous variables were assessed using analysis of variance (ANOVA) and ANOVA post hoc test or the Kruskal–Wallis test, while chi-square tests were used for categorical variables. A two-sided *p*-value < 0.05 was considered statistically significant.

The association between the HGI and overall mortality, cardiac mortality, and cardiovascular mortality in the general population was evaluated using multivariable Cox proportional hazards models. Given that the Q2 group exhibited the lowest relative risk, this group was selected as the reference group for the Cox regression analysis. Kaplan–Meier survival analysis was performed to compare survival outcomes across the HGI quartiles, with differences assessed using the log-rank test. Restricted cubic spline (RCS) curves were employed to explore potential nonlinear relationships between the HGI and the primary and secondary endpoints, including identifying inflection points. RCS is a statistical method that partitions the independent variable into multiple intervals and applies cubic polynomials within each segment to capture complex nonlinear trends. Finally, subgroup analyses were conducted using forest plots to evaluate the influence of confounding variables on the study outcomes, ensuring the robustness of the findings based on clinical expertise.

## 3. Results 

### 3.1 Characteristics of the Study Population Based on HGI Quartiles

This study analyzed data from 18,171 participants in the NHANES database, including 9044 males (48.99%) and 9127 females (51.01%). Participants were stratified into quartiles based on their HGI levels (Table 1).

**Table 1.** **Characteristics of participants in the NHANES data (1999–2018) by HGI quartile**.

Variables		Overall	Q1 (<–0.287)	Q2 (–0.287 to –0.028)	Q3 (–0.028 to 0.230)	Q4 (≥0.230)	*p*-value
		n = 18,171	n = 4535	n = 4540	n = 4552	n = 4544	
Age, years		46.99 (46.54, 47.45)	42.00 (30.00, 55.00)	44.00 (31.00, 57.00)	48.00 (35.00, 60.00)	54.00 (41.00, 65.00)	<0.0001
Sex, n (%)							<0.0001
	Male	9044 (48.99%)	2657 (58.65%)	2248 (47.83%)	2094 (43.93%)	2045 (42.99%)	
	Female	9127 (51.01%)	1878 (41.35%)	2292 (52.17%)	2458 (56.07%)	2499 (57.01%)	
BMI, kg/m^2^		28.72 (28.56, 28.89)	27.40 (24.00, 31.70)	27.22 (23.70, 31.38)	27.40 (23.86, 32.00)	28.74 (24.63, 33.98)	<0.0001
Race, n (%)							<0.0001
	Non–Hispanic White	8378 (69.73%)	2423 (75.66%)	2299 (72.58%)	2115 (69.59%)	1541 (57.36 %)	
	Mexican American	3190 (7.97%)	797 (7.84%)	904 (8.90%)	718 (6.89%)	771 (8.27%)	
	Non–Hispanic Black	3511 (10.73%)	594 (6.50%)	565 (6.67%)	907 (11.57%)	1445 (21.44%)	
	Other Hispanic	1549 (5.33%)	376 (4.79%)	386 (5.51%)	402 (5.56%)	385 (5.59%)	
	Other Races	1543 (6.23%)	345 (5.22%)	386 (6.35%)	410 (6.40%)	402 (7.34%)	
Marital, n (%)							<0.0001
	Married	9820 (57.25%)	2424 (56.20%)	2455 (57.33%)	2510 (58.31%)	2431 (57.31%)	
	Never married	3081 (17.35%)	892 (20.22%)	841 (18.80%)	728 (15.77%)	620 (13.19%)	
	Divorced	1842 (9.80%)	396 (8.40%)	445 (9.50%)	461 (10.52%)	540 (11.34%)	
	Living with partner	1376 (7.80%)	405 (9.00%)	363 (8.07%)	304 (6.87%)	304 (6.90%)	
	Widowed	1471 (5.48%)	286 (4.06%)	303 (4.30%)	386 (5.94%)	496 (8.62%)	
	Separated	581 (2.31%)	132 (2.12%)	133 (2.00%)	163 (2.60%)	153 (2.64%)	
Smoke, n (%)							0.2614
	Never	9820 (53.54%)	2426 (54.13%)	2492 (54.66%)	2458 (52.71%)	2444 (52.20%)	
	Former	8351 (46.46%)	2109 (45.87%)	2048 (45.34%)	2094 (47.29%)	2100 (47.80%)	
Alcohol use, n (%)							<0.0001
	Never	2523 (10.97%)	467 (8.51%)	574 (10.21%)	666 (11.05%)	816 (15.49%)	
	Former	3144 (14.30%)	649 (11.21%)	700 (13.00%)	797 (15.14%)	998 (19.53%)	
	Moderate	2685 (16.98%)	735 (17.60%)	741 (18.81%)	660 (16.34%)	549 (14.34%)	
	Mild	6249 (37.01%)	1533 (36.83%)	1567 (36.88%)	1623 (38.45%)	1526 (35.63%)	
	Heavy	3570 (20.74%)	1151 (25.86%)	958 (21.09%)	806 (19.01%)	655 (15.02%)	
Stroke, n (%)							<0.0001
	No	17,516 (97.25%)	4385 (97.56%)	4395 (97.42%)	4412 (97.79%)	4324 (95.89%)	
	Yes	655 (2.75%)	150 (2.44%)	145 (2.58%)	140 (2.21%)	220 (4.11%)	
FBG, mg/dL		104.13 (103.57, 104.70)	102.80 (96.00, 112.00)	98.00 (91.00, 105.00)	96.00 (90.00, 103.00)	97.00 (89.00, 109.30)	<0.0001
HbA1c, %		5.55 (5.53, 5.57)	5.10 (4.90, 5.30)	5.30 (5.10, 5.50)	5.50 (5.30, 5.70)	5.90 (5.60, 6.30)	<0.0001
ALT, U/L		25.44 (25.03, 25.85)	22.00 (17.00, 30.00)	21.00 (16.00, 28.00)	20.00 (16.00, 27.00)	21.00 (16.00, 28.00)	<0.0001
AST, U/L		24.91 (24.63, 25.19)	23.00 (19.00, 27.00)	22.00 (19.00, 26.00)	22.00 (19.00, 26.00)	23.00 (19.00, 27.00)	<0.0001
CR, mg/dL		0.88 (0.87, 0.88)	0.86 (0.72, 1.00)	0.82 (0.70, 0.98)	0.82 (0.70, 0.99)	0.83 (0.70, 1.00)	<0.0001
TG, mg/dL		120.32 (118.75, 121.89)	103.00 (70.00, 152.00)	102.00 (72.00, 149.00)	102.00 (71.00, 143.00)	109.00 (75.00, 160.00)	0.0001
TC, mg/dL		194.09 (193.19, 194.99)	186.00 (162.00, 213.00)	193.00 (167.00, 218.00)	195.00 (170.00, 223.00)	194.00 (166.00, 222.00)	<0.0001
HDL-C, mg/dL		54.05 (53.63, 54.47)	50.00 (42.00, 61.00)	52.00 (43.00, 63.00)	53.00 (44.00, 64.00)	51.00 (42.00, 62.00)	<0.0001
LDL-C, mg/dL		115.98 (115.23, 116.73)	109.00 (89.00, 134.00)	115.00 (93.00, 137.00)	116.00 (94.00, 141.00)	114.00 (90.00, 140.00)	<0.0001
SII		545.84 (538.56, 553.11)	466.71 (340.94, 652.50)	479.17 (352.80, 670.47)	479.29 (347.35, 664.71)	468.22 (328.91, 673.77)	0.7927
WBC, 10^9^/L		6.76 (6.71, 6.81)	6.40 (5.40, 7.60)	6.40 (5.40, 7.80)	6.40 (5.40, 7.70)	6.70 (5.50, 8.10)	<0.0001
LC, 10^9^/L		2.00 (1.98, 2.01)	1.90 (1.50, 2.30)	1.90 (1.50, 2.30)	1.90 (1.60, 2.30)	2.00 (1.60, 2.50)	<0.0001
NC, 10^9^/L		3.98 (3.94, 4.02)	3.70 (3.00, 4.70)	3.70 (3.00, 4.70)	3.70 (2.90, 4.70)	3.80 (2.90, 4.90)	0.0288
PLT, 10^9^/L		250.81 (249.24, 252.38)	245.00 (209.00, 285.00)	245.00 (209.00, 285.00)	249.00 (211.00, 292.00)	246.00 (207.00, 297.00)	<0.0001
HGI, %		–0.06 (–0.08, –0.05)	–0.48 (–0.63, –0.37)	–0.15 (–0.22, –0.09)	0.08 (0.02, 0.15)	0.41 (0.31, 0.60)	<0.0001
Hypertension, n (%)							<0.0001
	No	10,594 (63.72%)	2766 (65.55%)	2955 (69.39%)	2695 (64.01%)	2178 (52.86%)	
	Yes	7577 (36.28%)	1769 (34.45%)	1585 (30.61%)	1857 (35.99%)	2366 (47.14%)	
CHD, n (%)							<0.0001
	No	17,449 (96.63%)	4367 (96.96 %)	4383 (97.10 %)	4390 (97.02%)	4309 (94.98%)	
	Yes	722 (3.37%)	168 (3.04%)	157 (2.90%)	162 (2.98 %)	235 (5.02%)	
Angina, n (%)							<0.0001
	No	17,678 (97.70%)	4431 (98.05%)	4440 (98.18%)	4430 (97.90%)	4377 (96.26%)	
	Yes	493 (2.30%)	104 (1.95%)	100 (1.82%)	122 (2.10%)	167 (3.74%)	
CKD, n (%)							<0.0001
	No	15,030 (86.95%)	3847 (89.27%)	3897 (89.01%)	3834 (88.04%)	3452 (79.36%)	
	Yes	3141 (13.05%)	688 (10.73%)	643 (10.99%)	718 (11.96%)	1092 (20.64%)	
CHF, n (%)							<0.0001
	No	17,644 (97.82%)	4431 (98.50%)	4448 (98.46%)	4433 (98.04%)	4332 (95.65%)	
	Yes	527 (2.18%)	104 (1.50 %)	92 (1.54%)	119 (1.96%)	212 (4.35%)	
COPD, n (%)							<0.0001
	No	17,382 (95.81%)	4373 (96.76%)	4384 (96.47%)	4335 (95.59%)	4290 (93.78%)	
	Yes	789 (4.19%)	162 (3.24%)	156 (3.53%)	217 (4.41%)	254 (6.22%)	
Hyperlipidemia, n (%)							<0.0001
	No	4958 (28.85%)	1444 (33.37%)	1309 (30.18%)	1247 (28.43%)	958 (20.97%)	
	Yes	113,213 (71.15%)	3091 (66.63%)	3231 (69.82%)	3305 (71.57%)	3586 (79.03%)	
DM, n (%)							<0.0001
	No	11,968 (71.54%)	2723 (66.09%)	3366 (78.72%)	3378 (79.29%)	2501 (59.63%)	
	Yes	3301 (13.61%)	830 (13.00%)	441 (7.43%)	516 (8.59%)	1514 (29.47%)	
Cancer, n (%)							0.0006
	No	16,493 (90.85%)	4151 (91.99%)	4169 (91.36%)	4125 (90.54%)	4048 (88.88%)	
	Yes	1678 (9.15%)	384 (8.01%)	371 (8.64%)	427 (9.46%)	496 (11.12%)	
Outcomes, n (%)							
	All–cause mortality	2700 (10.67%)	645 (9.80%)	587 (8.89%)	656 (10.13%)	812 (15.10%)	<0.0001
	Cardiac mortality	710 (2.7%)	158 (2.23%)	135 (2.08%)	172 (2.52%)	245 (4.53%)	<0.0001
	Cardiovascular mortality	855 (4.7%)	196 (2.72%)	175 (2.65%)	205 (2.90%)	279 (5.10%)	<0.0001

Continuous variables are expressed as the median (Q1, Q3), and categorical variables are described by the n (%). Abbreviations: FBG, fasting blood–glucose; CR, creatinine.

Significant differences were observed across the HGI quartiles. Gender distribution revealed a trend whereby the proportion of females included in the higher HGI levels increased whereas the male participants decreased (females: Q1 = 1878 (41.35%), Q2 = 2292 (52.17%), Q3 = 2458 (56.07%), Q4 = 2499 (57.01%); males: Q1 = 2657 (58.65%), Q2 = 2248 (47.83%), Q3 = 2094 (43.93%), Q4 = 2045(42.99%)) (*p* < 0.05) (Table 1). The racial analysis showed that the proportion of non-Hispanic white individuals decreased, while non-Hispanic black individuals increased with higher HGI levels (Non-Hispanic white: Q1 = 2423 (75.66%), Q2 = 2299 (72.58%), Q3 = 2115 (69.59%), Q4 = 1541 (57.36%), Non-Hispanic black: Q1 = 594 (6.50%), Q2 = 565 (6.67%), Q3 = 907 (11.57%), Q4 = 1445 (21.44%)); other racial groups exhibited variable trends (*p* < 0.05; Table 1). Marital history analysis indicated that the proportions of divorced and widowed individuals increased with higher HGI levels, while other marital statuses fluctuated irregularly (*p* < 0.05; Table 1). Regarding alcohol consumption, the prevalence of individuals who never drank or were former drinkers was significantly higher in Q4 compared to Q1, whereas heavy drinkers were more prevalent in Q1 than in Q4; other categories showed inconsistent trends (*p* < 0.05; Table 1). The normality assessment demonstrated that the distributions of age (years), BMI, fasting blood–glucose (FBG), HbA1c, ALT, AST, CR, TG, TC, HDL-C, LDL-C, systemic immune-inflammatory index (SII), WBC, LC, NC, PLT, and HGI were not consistent with a normal distribution (**Supplementary Table 1**). Additionally, AST, CR, TG, and DM prevalence were significantly higher in Q1 and Q4 compared to Q2 and Q3 (*p* < 0.05; Table 1; **Supplementary Table 2**). Conversely, HDL-C, LDL-C, and SII were significantly higher in Q2 and Q3 compared to Q1 and Q4. Age, BMI, glycated hemoglobin, WBC, NC, and LC were significantly higher in Q4 than in Q1, Q2, and Q3. In contrast, ALT and FBG levels were highest in Q1, while PLT and TC peaked in Q3 (Table 1; **Supplementary Table 2**).

**Fig. 2.**
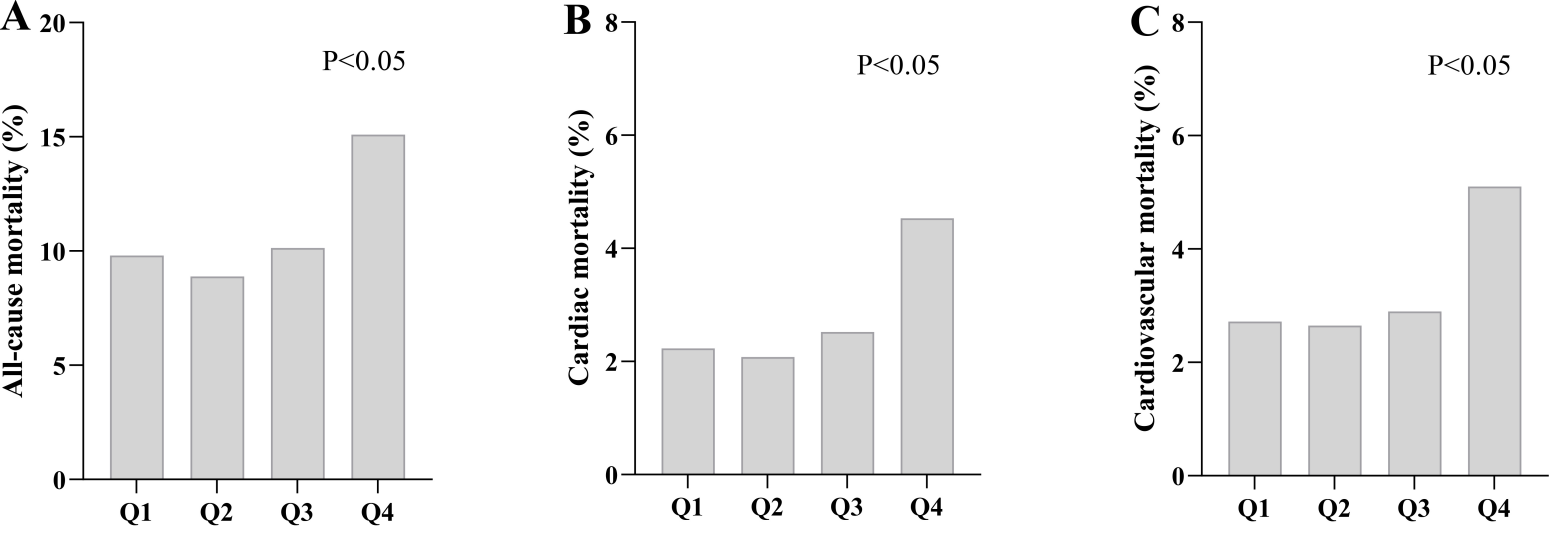
**Comparison of the incidence of cardiac events in the HGI quartile population**. Correlation between HGI and outcome events. (A) Comparison of all-cause mortality between groups based on HGI quartiles. (B) Comparison of cardiac mortality between groups based on HGI quartiles. (C) Comparison of cardiovascular mortality between groups based on HGI quartiles. Abbreviations: Q1, first quartile; Q2, second quartile; Q3, third quartile; Q4, fourth quartile.

The prevalence of smoking, hypertension, coronary heart disease (CHD), CKD, CHF, COPD, hyperlipidemia, DM, cancer, and stroke was significantly greater in Q4 compared to other quartiles (*p* < 0.05; Table 1). Regarding study outcomes, both all-cause mortality (primary endpoint) and cardiovascular-related mortality (secondary endpoints including cardiac mortality and cardiovascular mortality) exhibited a U-shaped trend across the HGI quartiles, with the highest rates observed in Q4 (*p* < 0.05; Table 1; Fig. 2; **Supplementary Table 2**).

### 3.2 Survival Analysis Based on HGI Quartiles

**Fig. 3.**
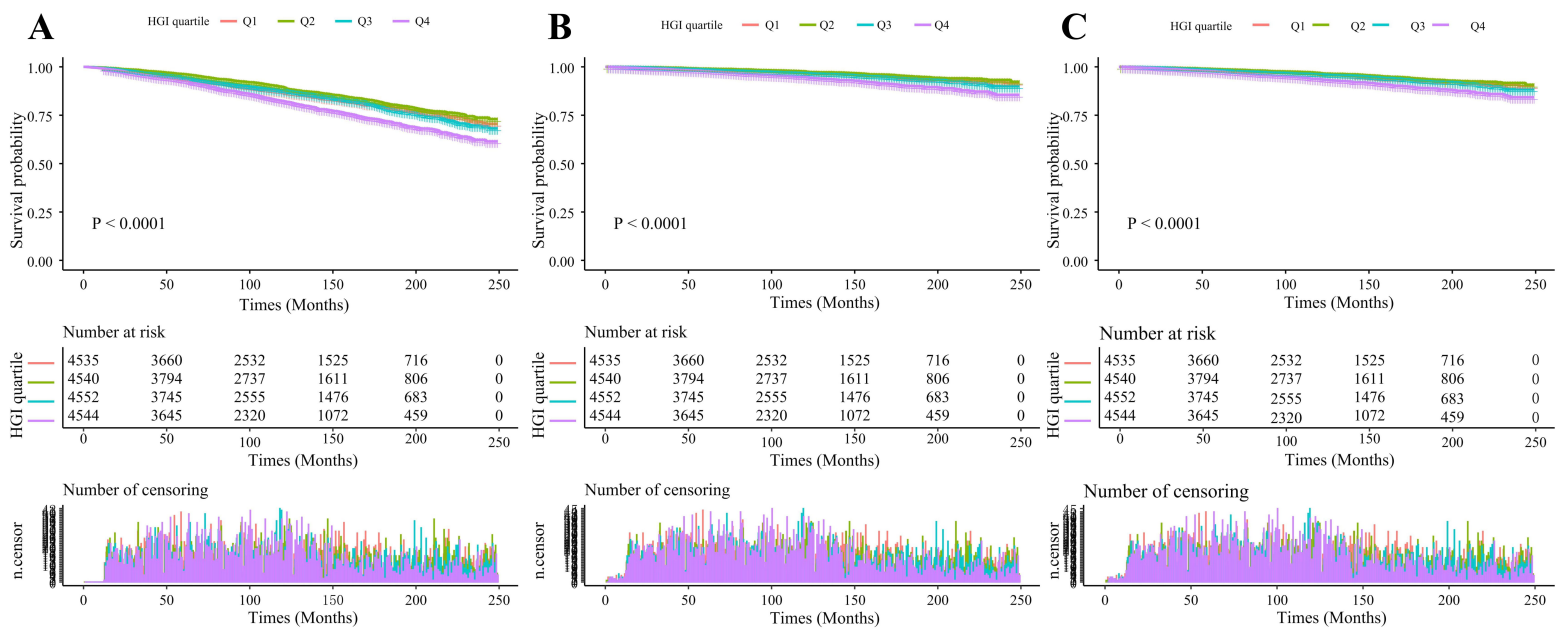
**Kaplan–Meier cumulative risk curve for cardiac events**. (A) All-cause mortality. (B) Cardiac mortality. (C) Cardiovascular mortality. Abbreviations: Q1, first quartile; Q2, second quartile; Q3, third quartile; Q4, fourth quartile.

This study had a median follow-up duration of 112 months. Our analysis revealed significantly higher incidence rates of all-cause mortality in the highest quartile (Q4) compared to Q1, Q2, and Q3 (*p* < 0.05). Similarly, we observed elevated cardiac and cardiovascular mortality rates in the Q4 group compared to lower quartiles (*p* < 0.05; Fig. 3).

### 3.3 Correlation of the HGI With Outcome Events

Using Cox proportional hazards models with Q2 as the reference category, we examined the association between the HGI and outcome events.

For all-cause mortality, both low HGI (Q1) (hazard ratio (HR) (95% confidence interval (CI)) = 1.269 (1.082, 1.488); *p *= 0.003) and high HGI (Q4) values (HR (95% CI) = 1.232 (1.065, 1.426); *p *= 0.005) were associated with increased risks, independent of adjusted risk factors (Table 2). The trend tests did not indicate a significant linear trend (*p* > 0.05), likely due to both low and high HGI values being risk factors for all-cause mortality.

**Table 2.** **The weighted multivariable Cox proportional hazards models between the HGI and different follow-up outcomes**.

Exposure			Non-adjusted		Adjust I		Adjust II	
			HR (95% CI)	*p*-value	HR (95% CI)	*p*-value	HR (95% CI)	*p*-value
All-cause mortality								
	HGI quartile							
		Q1	1.185 (1.036, 1.356)	0.013*	1.266 (1.102, 1.455)	0.001**	1.269 (1.082, 1.488)	0.003**
		Q2	1		1		1	
		Q3	1.214 (1.365, 1.080)	0.001**	0.999 (0.768, 1.123)	0.992	1.034 (0.901, 1.188)	0.634
		Q4	2.010 (1.759, 2.297)	<0.001***	1.303 (1.123, 1.511)	0.001**	1.232 (1.065, 1.426)	0.005**
	HGI for trend		1.240 (1.184, 1.298)	<0.001***	1.058 (1.008, 1.111)	0.022*	1.044 (0.997, 1.093)	0.067
Cardiac mortality								
	HGI quartile							
		Q1	1.150 (0.853, 1.550)	0.359	1.194 (0.884, 1.613)	0.248	1.163 (0.854, 1.584)	0.337
		Q2	1		1		1	
		Q3	1.300 (1.002, 1.687)	0.049	1.070 (0.825, 1.389)	0.609	1.092 (0.832, 1.434)	0.526
		Q4	2.608 (1.929, 3.527)	<0.001***	1.630 (1.181, 2.250)	0.003**	1.516 (1.100, 2.088)	0.011*
	HGI for trend		1.373 (1.245, 1.515)	<0.001***	1.154 (1.048, 1.271)	0.004**	1.131 (1.029, 1.243)	0.010*
Cardiovascular mortality								
	HGI quartile							
		Q1	1.204 (0.848, 1.428)	0.474	1.167 (0.900, 1.513)	0.241	1.147 (0.872, 1.509)	0.323
		Q2	1		1		1	
		Q3	1.168 (0.926, 1.473)	0.190	0.951 (0.759, 1.191)	0.664	0.984 (0.775, 1.251)	0.900
		Q4	2.289 (1.765, 2.969)	<0.001***	1.415 (1.072, 1.869)	0.014*	1.334 (1.013, 1.756)	0.039*
	HGI for trend		1.306 (0.765, 1.198)	<0.001***	1.093 (0.914, 1.004)	0.038*	1.077 (0.992, 1.170)	0.074

*p < 0.05, **p < 0.01, ***p < 0.001. The non-adjusted model adjusts for no characteristics. The adjusted I model adjusts for age, gender, BMI, and race. The adjusted II model adjusts for age, gender, BMI, race, marital, smoking, alcohol use, stroke, hypertension, CHD, angina, CKD, CHF, COPD, hyperlipidemia, cancer, ALT, AST, CR, SII, and WBC.

For cardiac mortality, high HGI (Q4) was a significant risk factor (HR (95% CI) = 1.516 (1.100, 2.088); *p* = 0.011), while low HGI (Q1) showed no significant association (*p* > 0.05). The trend tests confirmed that a high HGI value was a risk factor for cardiac mortality (HR (95% CI) = 1.131 (1.029, 1.243); *p* = 0.010; Table 2).

Regarding cardiovascular mortality, a high HGI (Q4) was significantly associated with increased risk (HR (95% CI) = 1.334 (1.013, 1.756); *p* = 0.039), whereas a low HGI (Q1) did not demonstrate clinical significance (*p* > 0.05; Table 2).

### 3.4 Threshold Effect Analysis of Three Different Follow-up Endpoints

**Fig. 4.**
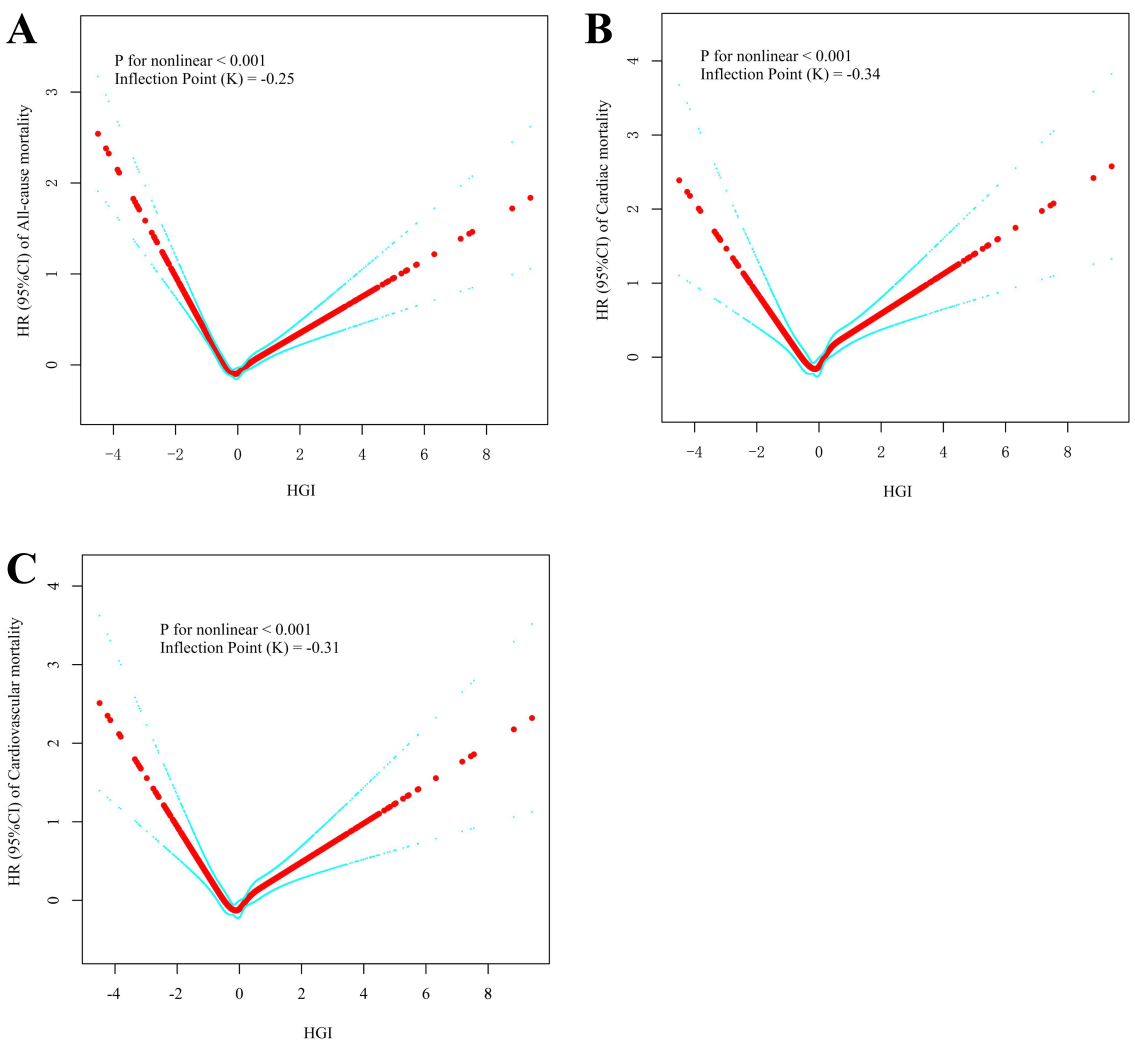
**Restricted cubic spline curve analysis for the HGI hazard ratio**. (A) All-cause mortality. (B) Cardiac mortality. (C) Cardiovascular mortality.

The RCS analyses revealed a U-shaped nonlinear relationship between the HGI and all-cause mortality (*p* for nonlinearity <0.001), with an inflection point at HGI = –0.25. Similarly, the U-shaped relationships were observed for cardiac mortality (*p* for nonlinearity <0.001, inflection point = –0.34) and cardiovascular mortality (*p* for nonlinearity < 0.001, inflection point = –0.31) (Table 3; Fig. 4).

**Table 3.** **Threshold effect analysis of three different follow-up endpoints**.

Standard linear regression model		All-cause mortality		Cardiac mortality		Cardiovascular mortality	
		β/OR (95% CI)	*p*-value	β/OR (95% CI)	*p*-value	β/OR (95% CI)	*p*-value
Two-stage regression models		1.01 (0.95, 1.09)	0.686	1.20 (1.07, 1.35)	0.001	1.12 (0.99, 1.25)	0.052
Inflection point (K)		–0.25		–0.34		–0.31	
	<K	0.54 (0.47, 0.61)	<0.001	0.54 (0.40, 0.71)	<0.001	0.52 (0.41, 0.66)	<0.001
	>K	1.23 (1.15, 1.31)	<0.001	1.37 (1.23, 1.52)	<0.001	1.30 (1.17, 1.44)	<0.001
log-likelihood ratio test			<0.001		<0.001		<0.001

Adjusted for age, gender, BMI, race, marital history, smoking, alcohol use, stroke, hypertension, CHD, angina, CKD, CHF, COPD, hyperlipidemia, cancer, ALT, AST, CR, SII, and WBC. OR, odds ratio.

### 3.5 Subgroup Analysis

**Fig. 5.**
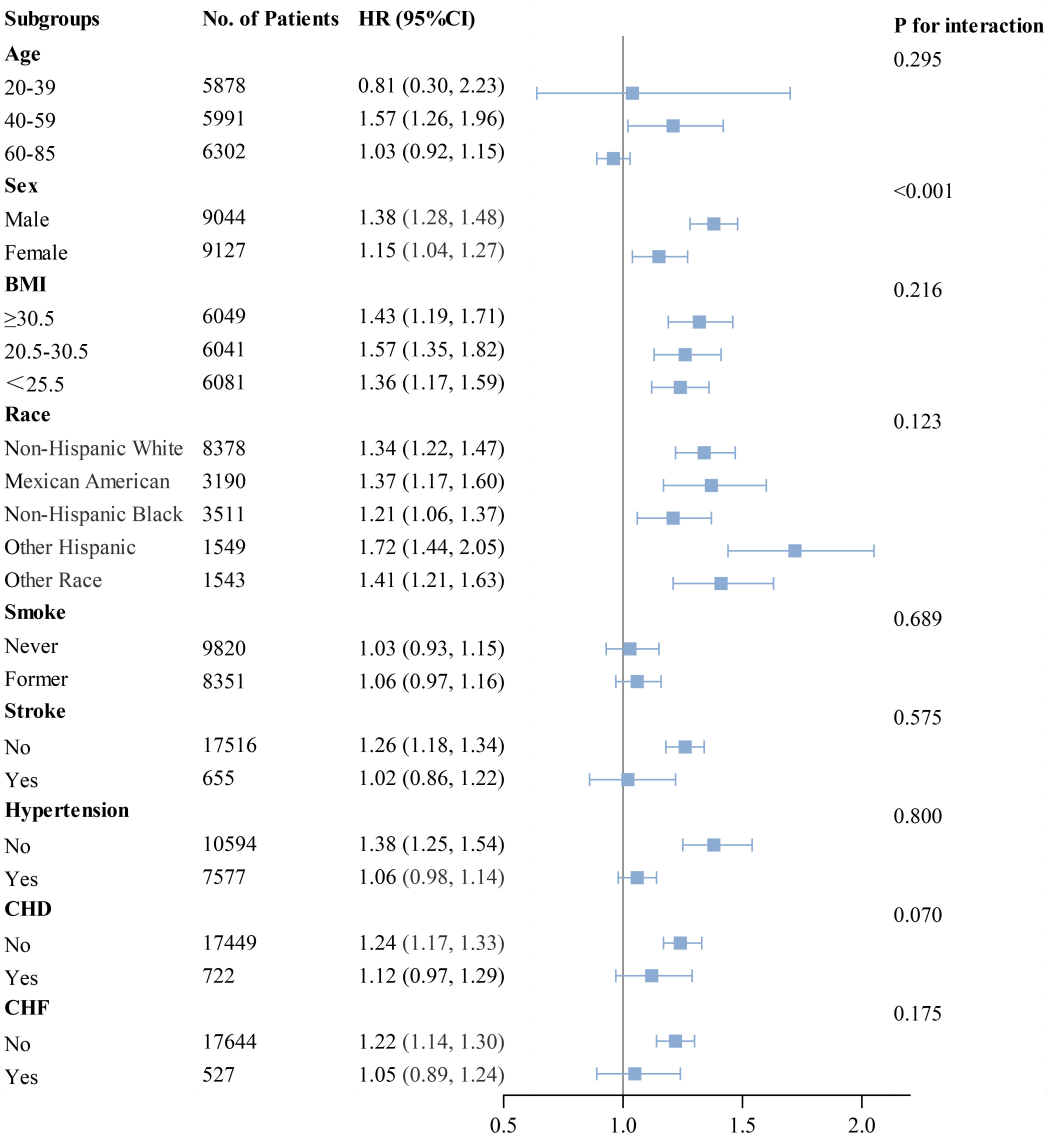
**Subgroup analysis of outcomes from all-cause mortality**.

**Fig. 6.**
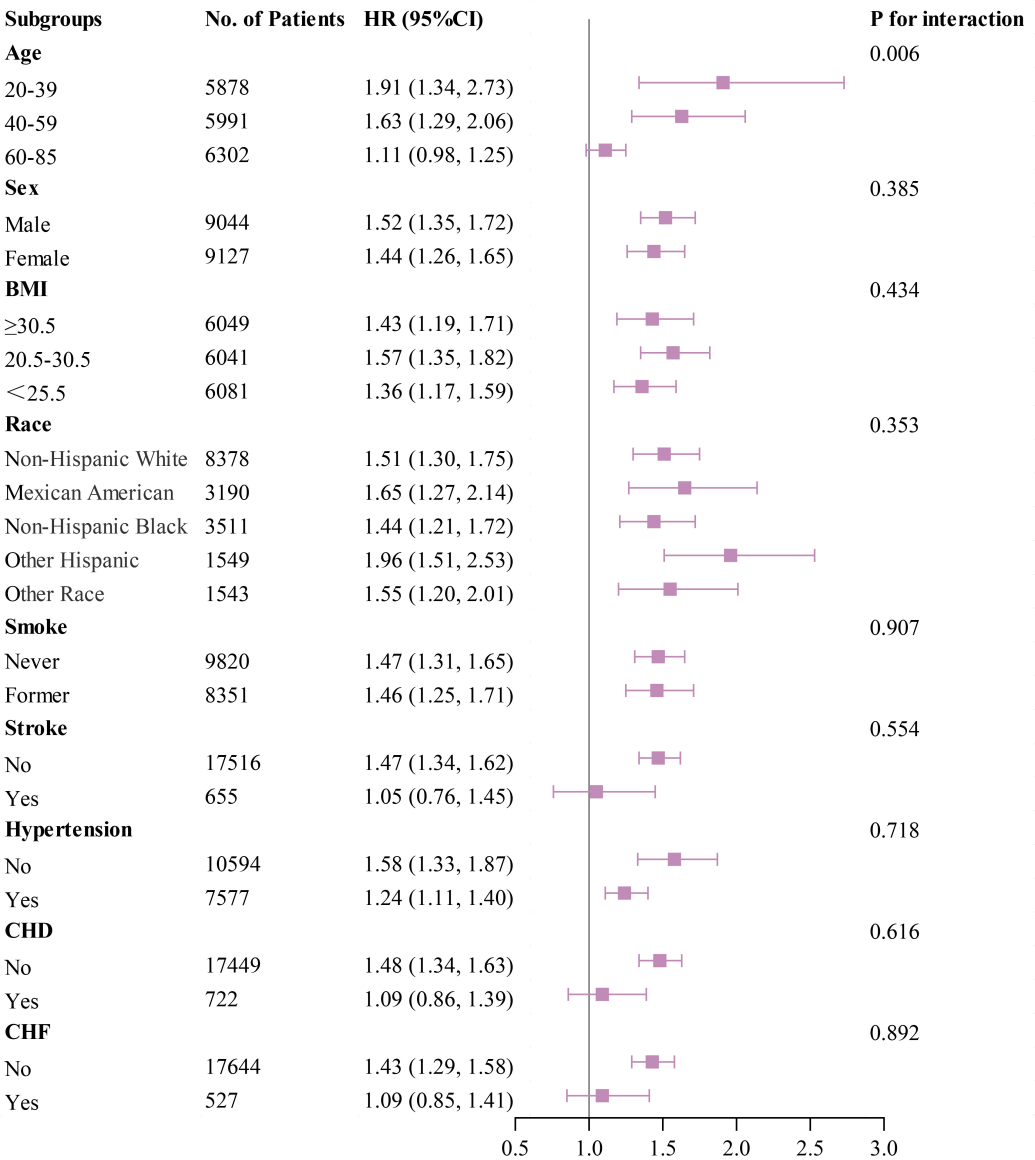
**Subgroup analysis of outcomes from cardiac mortality**.

**Fig. 7.**
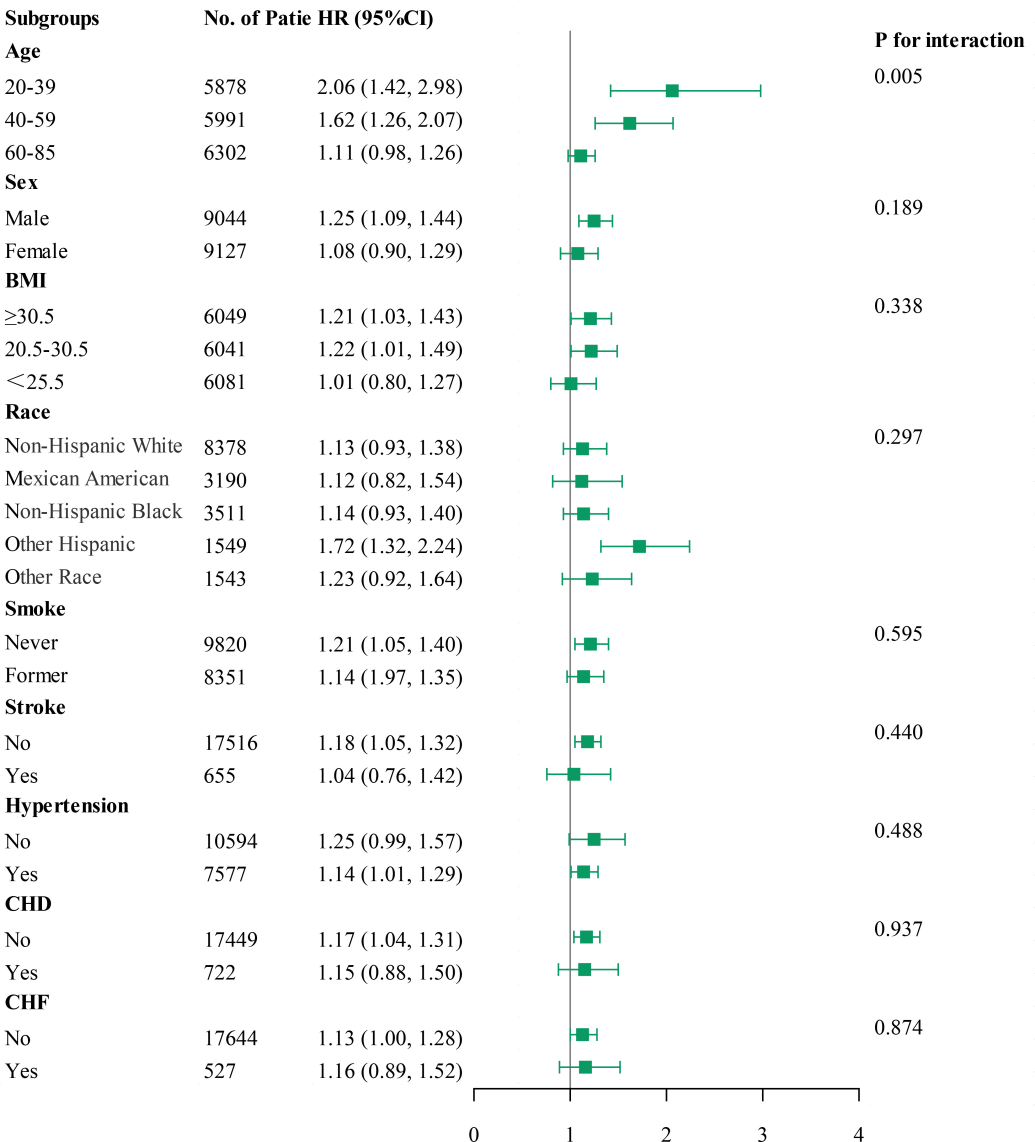
**Subgroup analysis of outcomes from cardiovascular mortality**.

Subgroup analyses were performed using forest plots to evaluate the robustness of study outcomes and assess whether the HGI differs as a common risk factor in all-cause mortality, cardiac mortality, and cardiovascular mortality. Subgroup analyses for outcome events were conducted based on sex, age, BMI, race, smoking status, history of stroke, hypertension, CHD, and CHF. Our findings revealed that among groups with all-cause mortality as the primary endpoint, except for sex, elevated HGI values were closely associated with higher rates of all-cause mortality in all other subgroups (Fig. 5). In the subsequent Cox regression analysis stratified by gender, both Q1 and Q4 were found to be associated with all-cause mortality in males. In contrast, in females, only Q1 was related to all-cause mortality (**Supplementary Table 3**). Moreover, in groups where cardiac mortality and cardiovascular mortality were endpoints, except for age, an elevated HGI was consistently associated with adverse outcomes in the general population (Figs. 6,7). In the subsequent Cox analysis stratified by age subgroups, the following associations were observed: For cardiac mortality, Q3 and Q4 were identified as significant risk factors in the 20–39 age group, whereas no significant associations were found across Q1–Q4 in the 40–59 age group. In the 65–85 age group, Q1 and Q4 demonstrated significant associations with cardiac mortality (**Supplementary Table 4**). Regarding cardiovascular mortality, Q3 and Q4 showed significant associations in the 20–39 age group, while no significant relationships were observed across Q1–Q4 in the 40–59 age group; meanwhile, in the 65–85 age group, Q1 and Q4 were significantly associated with cardiovascular mortality (**Supplementary Table 4**).

## 4. Discussion

This study evaluated the associations between the HGI and all-cause mortality, cardiac mortality, and cardiovascular mortality in the general population. The results revealed a U-shaped relationship between the HGI and all-cause mortality, with high and low HGI levels associated with increased mortality rates. However, only high HGI values were significantly associated with increased cardiac and cardiovascular mortalities, while low HGI values showed no significant statistical relevance. This suggests that while both high and low HGI values may independently contribute as risk factors for all-cause mortality in the general population, only high HGI values emerge as an independent risk factor for cardiac mortality and cardiovascular mortality.

The HGI, introduced by Hempe *et al*. [6] in 2002, offers a novel approach to quantifying discrepancies between individual A1c levels and average blood glucose levels. Unlike FPG and HbA1c, elevated HGI values correlate with increased diabetes risk independent of current blood glucose levels. Previous research has demonstrated the congruence of continuous glucose monitoring (CGM) with the HGI, underscoring its reliability in assessing blood glucose status [12]. A study has also indicated that as populations transition from low to high HGI subgroups, average HbA1c levels rise despite decreasing average FPG levels, suggesting that postprandial glucose fluctuations do not significantly impact variations in the HGI [13]. Thus, high HGI values may signify long-term blood glucose stability. Clinically, discrepancies between HbA1c levels and actual blood–glucose control are common due to glycemic gaps. Consequently, patients with similar mean blood–glucose levels can exhibit substantial differences in HbA1c levels, potentially leading clinicians to overlook these gaps and misjudge therapeutic strategies, ultimately compromising patient treatment [14]. In contrast to HbA1c, utilizing the HGI for patient blood–glucose assessments may offer superior benefits in clinical management, mitigating the risk of therapeutic misjudgments [15]. Considering that HbA1c is currently the widely recognized indicator for blood–glucose control analysis and the challenges of implementing the HGI in clinical practice, future research may need to explore further the similarities and differences between the HGI and HbA1c. Randomized controlled trials guided by HGI thresholds (e.g., implementing differentiated glycemic targets for patients with high HGI values) are also required to develop decision support systems that dynamically integrate HGI, CGM data, and machine learning algorithms to balance blood–glucose control with hypoglycemia risk.

In the two decades since the HGI was proposed, this index has continued to be a subject of active research. The HGI has been established as a predictor of diabetes onset and complications such as diabetic nephropathy and CVD in diabetic patients [16, 17, 18, 19, 20]. Moreover, studies have demonstrated a direct association between elevated HGI values and metabolic syndrome risk in older populations [9, 21]. Higher HGI levels have also proven effective in identifying susceptibility to non-alcoholic fatty liver disease and hypertension among individuals with diabetes [22, 23, 24, 25]. Regarding CVDs, the HGI reliably predicts cardiovascular risk in diabetic populations [26, 27, 28, 29, 30, 31, 32]. Large-scale clinical trials, including the DEVOTE trial, have notably linked elevated HGI levels in type 2 diabetes mellitus (T2DM) patients to increased risks of major adverse cardiovascular events over extended follow-up periods [33]. Similarly, findings from the Ale-Cardio trial highlighted a 16% heightened risk of cardiovascular death per percentage point increase in the HGI [34]. Meta-analyses have further confirmed elevated HGI levels as significantly associated with heightened risks of cardiovascular complications and overall mortality in people with T2DM [20]. Despite these insights, most studies have been cross-sectional and confined to specific diabetic populations, often lacking long-term follow-ups. This limits definitive conclusions about broader prognostic implications of the HGI across the general population. Additionally, associations between the HGI and metabolic and cardiovascular outcomes appear applicable beyond diabetes [35]. A recent study demonstrated a correlation between increased HGI values and telomere attrition. As the HGI levels rose, so did telomere wear, indicating potential impacts on lifespan [28]. These findings suggest that the HGI may be an independent risk factor for all-cause mortality in the general population. Our study findings support this conclusion. In our investigation, individuals with elevated HGI values showed significantly higher inflammatory and metabolic markers, including BMI, WBC, and HbA1c. Moreover, compared to those with lower HGI values, these participants exhibited increased risks of age-related conditions such as hypertension and CVDs. Importantly, the high HGI group also demonstrated markedly elevated risks of all-cause mortality, cardiac mortality, and cardiovascular mortality compared to the low HGI group, aligning with prior research hypotheses.

Recent studies have highlighted that high and low HGI values can influence patient outcomes differently. For instance, a cohort study by Østergaard *et al*. [36] involving 1910 patients with T2DM suggested that low HGI values might increase the risk of myocardial infarction in patients with CHD. Pan *et al*. [32] also observed a U-shaped relationship between the HGI value and one-year stroke risk, indicating that low and high HGI levels are associated with adverse cerebrovascular outcomes. A prospective study of 5260 critically ill patients with CHD admitted to the ICU found that both high and low HGI values were significantly linked to negative outcomes at 30 days and 365 days [37]. However, the association between a low HGI and cardiovascular prognosis in the general population remains contentious. This study suggests that the correlation analysis between a low HGI and death from cardiac disease and cardiovascular mortality did not reach statistical significance; however, the *p*-values for these associations were close to 0.05. This indicates a potential risk that cannot be discounted outright. However, potential biases cannot be neglected, such as selection bias due to missing data, which may not fully represent the entire population. Moreover, the limited types of variables studied might have omitted important risk factors affecting outcomes and risk assessment stability.

The impact of HGI on outcome variables in the subgroup analysis conducted in this study remained consistent across predefined subgroups, except for differences related to sex in all-cause mortality and age in cardiac and cardiovascular mortalities. The observed sex disparity in all-cause mortality may reflect inherent differences in life expectancy between genders. Regarding cardiac mortality and cardiometabolic mortality, individuals aged 20–39 exhibited the highest mortality risk. The better self-care capabilities and perceived notions of being healthy may contribute to younger adults often underestimating their susceptibility to metabolic and CVDs such as hypertension, DM, and CHD. Clinical observations indicate that early-onset cardiovascular and metabolic diseases among younger adults can lead to poorer prognoses compared to older adults. Additionally, the forest plot suggests that patients without stroke, hypertension, coronary heart disease, or CHF but with high HGI values may have a higher mortality rate. This phenomenon is surprising, and to some extent, it is possible that the HGI is more closely associated with mortality in this group of patients. However, due to the retrospective cohort design of this study, study bias may exist. Therefore, further prospective studies may be needed in the future to explore this aspect.

This 20-year cohort study examines the association between the HGI and the risk of all-cause mortality, cardiac mortality, and cardiovascular mortality in the general population. Notably, this study marks the first documentation of the association between HGI values and cardiovascular mortality in the general population. In clinical settings, healthcare providers can assess the risk of cardiovascular mortality based on HGI levels, enabling timely intervention for high-risk patients.

## 5. Limitations 

This study has several limitations. Firstly, the study draws upon public data from the NHANES database, primarily derived from sampled questionnaire surveys of the general population, inherently introducing population data biases. Secondly, inherent relative errors and biases persist. Therefore, future prospective studies involving larger and more diverse populations should explore the association between HGI values and outcomes such as all-cause mortality, cardiac mortality, and cardiometabolic mortality. Lastly, given the many factors influencing mortality in the general population, not all relevant risk factors could be addressed in this study, necessitating further refinement and enhancement of its findings.

## 6. Conclusion

This study demonstrates that the HGI is associated with all-cause mortality, cardiac mortality, and cardiovascular mortality in the general population. Elevated HGI values emerged as a risk factor for all-cause mortality and cardiovascular mortality in this population, whereas a lower HGI level was linked solely to the general population.

## Data Availability

The data generated in this study are available upon request from the corresponding author.

## Abbreviations

HGI, hemoglobin glycation index; HbA1c, glycosylated hemoglobin; NHANES, National Health and Nutrition Examination Survey; RCS, restricted cubic spline; CVD, cardiovascular disease; DM, diabetes mellitus; NCHS, National Center for Health Statistics; BMI, body mass index; ALT, alanine aminotransferase; AST, aspartate aminotransferase; CR, creatinine; BUN, blood urea nitrogen; TG, triglycerides; TC, total cholesterol; HDL-C, high-density lipoprotein cholesterol; LDL-C, low-density lipoprotein cholesterol; UA, uric acid; FPG, fasting plasma glucose; WBC, white blood cell count; NC, neutrophil count; LC, lymphocyte count; PLT, platelet count; RBC, red blood cell count; CAD, coronary artery disease; CHF, congestive heart failure; COPD, chronic obstructive pulmonary disease; CKD, chronic kidney disease; CGM, continuous glucose monitoring; T2DM, type 2 diabetes mellitus.

## Supplementary Material

Supplementary material associated with this article can be found, in the online version, at https://doi.org/10.31083/RCM36792.
