# Supplementary material for: Association of Hemoglobin Glycation Index With All-Cause Mortality, Cardiac Mortality, and Cardiovascular Mortality in the General Population: A Retrospective Cohort Study of NHANES Data
Source: Rev Cardiovasc Med. 2025 Jul 28;26(7):36792. doi: 10.31083/RCM36792 (PMC12326456; doi:10.31083/RCM36792)
Supplement: Supplementary file 1 [file 2153-8174-26-7-36792-s1.docx]

Supplementary Materials

Supplement Table 1 Lilliefors (Kolmogorov-Smirnov) Normality Test

Supplement Table 2 Post-Hoc Tests for Analysis of Variance (ANOVA)

Supplement Table 3 Cox Regression Analysis of the Association Between HGI and All-Cause Mortality, Stratified by Gender

Supplement Table 4 Cox Regression Analysis of the Association Between HGI and Cardiac Mortality as well as Cardiovascular Mortality, Stratified by Age

Supplement Table 1 Lilliefors (Kolmogorov-Smirnov) Normality Test

| Variables | P - value |
| --- | --- |
| Age, years | <0.001 |
| BMI, kg/m2 | <0.001 |
| FBG, mg/dL | <0.001 |
| HbA1c, % | <0.001 |
| ALT, U/L | <0.001 |
| AST, U/L | <0.001 |
| CR, mg/dL | <0.001 |
| TG, mg/dL | <0.001 |
| TC, mg/dL | <0.001 |
| HDL-C, mg/dL | <0.001 |
| LDL-C, mg/dL | <0.001 |
| SII | <0.001 |
| WBC, 10^9^/L | <0.001 |
| LC, 10^9^/L | <0.001 |
| NC, 10^9^/L | <0.001 |
| PLT, 10^9^/L | <0.001 |
| HGI, % | <0.001 |

Abbreviations: BMI body mass index, FBG fasting blood-glucose, HbA1c glycohemoglobin, ALT alanine transaminase, AST aspartate transaminase, CR creatinine, TG triglyceride, TC Total cholesterol lipoprotein, HDL-C high density lipoprotein cholesterol, LDL-C low density lipoprotein cholesterin, SII systemic immune inflammatory index, WBC white blood cell count, LC lymphocyte count, NC neutrophils count, PLT platelets count, HGI hemoglobin glycation index.

Supplement Table 2 Post-Hoc Tests for Analysis of Variance (ANOVA)

| Variables | P-Value Q1-Q2 | P-Value Q1-Q3 | P-Value Q1-Q4 | P-Value Q2-Q3 | P-Value Q2-Q4 | P-Value Q3-Q4 |
| --- | --- | --- | --- | --- | --- | --- |
| Age, years | 0.122 | <0.001 | <0.001 | <0.001 | <0.001 | <0.001 |
| BMI, kg/m2 | 0.253 | 0.942 | <0.001 | 0.075 | <0.001 | <0.001 |
| FBG, mg/dL | <0.001 | <0.001 | <0.001 | 0.035 | <0.001 | <0.001 |
| HbA1c, % | <0.001 | <0.001 | <0.001 | <0.001 | <0.001 | <0.001 |
| ALT, U/L | 0.018 | <0.001 | 0.005 | 0.138 | 0.973 | 0.313 |
| AST, U/L | 0.004 | 0.002 | 0.633 | 0.996 | 0.121 | 0.070 |
| CR, mg/dL | 0.024 | 0.010 | 0.037 | 0.993 | <0.001 | <0.001 |
| TG, mg/dL | 0.453 | <0.001 | 0.999 | 0.047 | 0.486 | <0.001 |
| TC, mg/dL | <0.001 | <0.001 | <0.001 | 0.033 | 0.965 | 0.110 |
| HDL-C, mg/dL | 0.059 | <0.001 | 0.159 | 0.005 | 0.973 | 0.001 |
| LDL-C, mg/dL | <0.001 | <0.001 | <0.001 | 0.045 | 0.998 | 0.073 |
| SII | 0.979 | 0.659 | 0.180 | 0.877 | 0.366 | 0.819 |
| WBC, 109/L | 0.9322 | 0.323 | <0.001 | 0.690 | 0.001 | 0.044 |
| LC, 109/L | 0.518 | <0.001 | <0.001 | 0.009 | <0.001 | 0.161 |
| NC, 109/L | 0.955 | 0.352 | 0.998 | 0.670 | 0.986 | 0.454 |
| PLT, 109/L | <0.001 | <0.001 | <0.001 | 0.032334 | 0.0175 | 0.997 |
| HGI, % | <0.001 | <0.001 | <0.001 | <0.001 | <0.001 | <0.001 |

Abbreviations: BMI body mass index, FBG fasting blood-glucose, HbA1c glycohemoglobin, ALT alanine transaminase, AST aspartate transaminase, CR creatinine, TG triglyceride, TC Total cholesterol lipoprotein, HDL-C high density lipoprotein cholesterol, LDL-C low density lipoprotein cholesterin, SII systemic immune inflammatory index, WBC white blood cell count, LC lymphocyte count, NC neutrophils count, PLT platelets count, HGI hemoglobin glycation index.

Supplement Table 3 Cox Regression Analysis of the Association Between HGI and All-Cause Mortality, Stratified by Gender

| Exposure | Non-adjusted |  | Adjust I |  | Adjust II |  |
| --- | --- | --- | --- | --- | --- | --- |
| HGI quartile | HR (95%CI) | P - value | HR (95%CI) | P-value | HR (95%CI) | P-value |
| All-cause mortality |  |  |  |  |  |  |
| Male (n=2545) |  |  |  |  |  |  |
| Q1 | 1.115 (0.941,1.320) | 0.208 | 1.233 (1.047,1.454) | 0.012 | 1.210 (1.017,1.439) | 0.031 |
| Q2 | 1 |  | 1 |  | 1 |  |
| Q3 | 1.295 (1.083,1.548) | 0.004 | 1.122 (0.944,1.334) | 0.191 | 1.090 (0.914,1.298) | 0.338 |
| Q4 | 2.126 (1.743,2.594) | <0.001 | 1.568 (1.288,1.909) | <0.001 | 1.426 (1.175,1.729) | <0.001 |
| Female (n=1155) |  |  |  |  |  |  |
| Q1 | 1.236 (0.983,1.553) | 0.070 | 1.366 (0.732,1.064) | 0.014 | 1.364 (1.005,1.851) | 0.047 |
| Q2 | 1 |  | 1 |  | 1 |  |
| Q3 | 1.153 (0.946,1.407) | 0.159 | 0.872 (1.147,0.709) | 0.193 | 0.949 (0.746,1.207) | 0.669 |
| Q4 | 1.944 (1.606,2.352) | <0.001 | 1.080 (0.926,0.877) | 0.469 | 1.052 (0.825,1.340) | 0.684 |

Abbreviations: HR, hazard ratio; CI, confidence interval.

Supplement Table 4 Cox Regression Analysis of the Association Between HGI and Cardiac Mortality as well as Cardiovascular Mortality, Stratified by Age

| Exposure | Non-adjusted |  | Adjust I |  | Adjust II |  |
| --- | --- | --- | --- | --- | --- | --- |
| HGI quartile | HR (95%CI) | P - value | HR (95%CI) | P-value | HR (95%CI) | P -value |
| **Cardiac mortality** |  |  |  |  |  |  |
| 20-39 (n=23) |  |  |  |  |  |  |
| Q1 | 0.823 (0.147,4.619) | 0.825 | 0.688 (0.117,4.032) | 0.679 | 0.829 (0.261,2.636) | 0.751 |
| Q2 | 1 |  | 1 |  | 1 |  |
| Q3 | 5.047 (1.020,24.964) | 0.047 | 4.719 (0.976,22.822) | 0.054 | 5.235 (2.084,13.148) | <0.001 |
| Q4 | 8.264 (1.937,35.260) | 0.004 | 7.376 (1.778,30.600) | 0.006 | 8.190 (3.868,17.341) | <0.001 |
| 40-59 (n=96) |  |  |  |  |  |  |
| Q1 | 0.778 (0.360,1.682) | 0.524 | 0.685 (0.323,1.451) | 0.323 | 0.700 (0.335,1.463) | 0.344 |
| Q2 | 1 |  | 1 |  | 1 |  |
| Q3 | 0.866 (0.453,1.655) | 0.663 | 0.831 (0.426,1.622) | 0.587 | 0.845 (0.428,1.669) | 0.627 |
| Q4 | 1.818 (0.999,3.309) | 0.050 | 1.403 (0.742,2.652) | 0.297 | 1.038 (0.567,1.901) | 0.905 |
| 60-85 (n-591) |  |  |  |  |  |  |
| Q1 | 1.466 (1.101,1.953) | 0.009 | 1.378 (1.039,1.828) | 0.026 | 1.401 (1.041,1.885) | 0.026 |
| Q2 | 1 |  | 1 |  | 1 |  |
| Q3 | 1.057 (0.785,1.424) | 0.713 | 1.092 (0.809,1.473) | 0.566 | 1.144 (0.853,1.535) | 0.369 |
| Q4 | 1.576 (1.133,2.193) | 0.007 | 1.730 (1.239,2.416) | 0.001 | 1.582 (1.137,2.203) | 0.007 |
| **Cardiovascular mortality** |  |  |  |  |  |  |
| 20-39 (n=23) |  |  |  |  |  |  |
| Q1 | 0.823 (0.147,4.619) | 0.825 | 0.688 (0.117,4.032) | 0.679 | 0.829 (0.261,2.636) | 0.751 |
| Q2 | 1 |  | 1 |  | 1 |  |
| Q3 | 5.047 (1.020,24.964) | 0.047 | 4.719 (0.976,22.822) | 0.054 | 5.235 (2.084,13.148 ) | <0.001 |
| Q4 | 8.264 (1.937,35.260) | 0.004 | 7.376 (1.778,30.600) | 0.006 | 8.189 (3.868,17.341 ) | <0.001 |
| 40-59 (n=105) |  |  |  |  |  |  |
| Q1 | 0.688 (0.323,1.463) | 0.331 | 0.621 (0.299,1.288) | 0.200 | 0.617 (0.293,1.230 ) | 0.204 |
| Q2 | 1 |  | 1 |  | 1 |  |
| Q3 | 0.794(0.437,1.441) | 0.448 | 0.762(0.414, 1.401) | 0.382 | 0.756(0.401, 1.424) | 0.387 |
| Q4 | 1.480(0.839, 2.612) | 0.176 | 1.151(0.631,2.099) | 0.646 | 0.872(0.490, 1.552) | 0.642 |
| 60-85 (n=727) |  |  |  |  |  |  |
| Q1 | 1.414(1.099,1.820) | 0.007 | 1.347(1.048,1.732) | 0.020 | 1.391(1.070,1.808) | 0.014 |
| Q2 | 1 |  | 1 |  | 1 |  |
| Q3 | 0.950(0.739, 1.222) | 0.689 | 0.969(0.752,1.247) | 0.804 | 1.029(0.806,1.314) | 0.820 |
| Q4 | 1.406(1.048, 1.887) | 0.023 | 1.526(1.136,2.049) | 0.005 | 1.413(1.064, 1.876) | 0.017 |

Abbreviations: HR, hazard ratio; CI, confidence interval.
